# European study of research and development in mobility technology for persons with disabilities

**DOI:** 10.1186/1743-0003-9-23

**Published:** 2012-04-20

**Authors:** Brian Caulfield, Ted A Conway, Silvestro Micera

**Affiliations:** 1 University College Dublin, Dublin, Ireland; 2 GARDE Program Director, National Science Foundation, Arlington, VA, USA; 3 Ecole Polytechnique Federale de Lausanne, Lausanne (Switzerland) - Scuola Superiore Sant' Anna, Pisa, Italy

## Abstract

In the fall of 2010, the National Science Foundation, the National Institutes of Health and the U.S. Veteran's Administration jointly supported a review of mobility technology in Europe. A delegation of American Scientists traveled to Europe to visit a number of research centers and engaged in a demonstration and dialogue related to the global state-of-the-art for mobility impairment rectification and augmentation. From the observations and exchanges between the U.S. delegation and host institutions, the researchers were able to derive a series of papers which are now published in this thematic series of Journal of NeuroEngineering and Rehabilitation. The papers describe the main themes of the European mobility technology research activities showing a healthy picture of research and innovation in the field.

## Introduction

According to the McNeil Report and, more recently, the 2004 Louis Harris Poll [1] approximately 20% of the United States Population is experiencing some form of disability, as defined by the ADA Amendments Act of 2008 [2]. These disabilities are generally caused by trauma, degenerative diseases or simple aging. Accordingly, if a person lives long enough, she/he will become disabled in at least one major life function. A significant portion of the disability community includes those individuals experiencing some form of mobility impairment.

In 1979, Congress recognized the need to provide research that would address enabling and disability augmentation issues for an important group within the U.S. population. In House Report 95-993, the Committee of Science and Technology directed the National Science Foundation (NSF) to create a program designed to support basic and applied research directed at providing enabling technology for persons with disabilities. At the inception of the NSF 'Handicapped Research Program', the program would "... support research on the use of scientific and engineering development to improve (disabilities), and also to find ways to overcome locomotion and manipulatory limitations." [3] This program was charged with providing an interdisciplinary environment that included all of the basic engineering disciplines along with the emerging field of biomedical engineering.

Over the years, the 'Handicapped Research Program' has experienced a variety of name changes that reflect the evolution of societal needs. The current form of this program is the 'General & Age Related Disabilities Engineering' (GARDE) Program and provides funding for basic research that targets the general disability community as well as the ever increasing needs of an aging population. This current form of the program is directly in line with the NSF Strategic Plan for Fiscal Years 2011-2016 - Empowering the Nation Through Discovery and Innovation with the vision to "... capitalize on new concepts in science and engineering and provide global leadership in advancing research and education." [4] The GARDE Program supports basic research in the development of assistive technologies (e.g. mobility technologies) that enhances access to the creativity, problem solving skills and perseverance traits that are found throughout the community of persons with disabilities. By supporting engineering applications to minimize the effects of disabilities related issues, the U.S. benefits from the increased participation of this significant but highly underrepresented group in all areas of research, including science, technology, engineering and mathematics.

## Thematic series

A study of mobility technology research in Europe was jointly supported by the National Science Foundation, the National Institutes of Health and the U.S. Veteran's Administration. A delegation of research leaders (some with first-hand knowledge of mobility impairments) in the U.S. along with members of the World Technology Evaluation Corporation (WTEC) were tasked with identifying top researchers and institutions in a variety of countries within the European Union. Subsequent to this identification process, the research team, along with the GARDE Program Director and members of WTEC traveled to these top sites to engage in a demonstration and dialogue related to the global state-of-the-art for mobility impairment rectification and augmentation. The observations and exchanges between the U.S. delegation and host institutions provided a venue to identify and minimize the potential for research duplication ("reinventing the wheel") and opportunities for collaboration between U.S. researchers in mobility technology and their European counterparts. A detailed description of these interactions follows in subsequent articles of this special thematic series of the Journal of NeuroEngineering and Rehabilitation.

The papers that describe the main themes of European mobility technology research activities show a healthy picture of research and innovation in the field. However, that is not to say that improvements in approach and infrastructure are not required. Recent years have witnessed an explosion in the potential for advances in technologies designed to assist and enhance mobility. We have seen an increase in the availability and a reduction in the cost of biomedical sensor platforms, smart fabrics, robotic and neuro-prosthetic devices, social media platforms, and mobile and cloud computing platforms. This greatly increases the range of tools we can use to monitor, characterize, and enhance human behavior and mobility. However, this also presents a great challenge as there is a danger that we can become overwhelmed by the technological capabilities that are available to us and lose sight of what we mean to achieve for patients using these technology platforms. Having access to more technology tools is not simply enough to achieve advances that will make a meaningful and positive impact on the lives of the patients who need help. Real advances in this area will only take place when we place an emphasis on patient or user centered design, implementation and evaluation. This means we need to move away from a technology focused paradigm and bring different skill sets to bear on the problem. Thankfully, we are seeing the emergence of new approaches to the field with collaborations between clinicians, technologists and social scientists working towards a truly user centered model that should enhance our capability for advancement in the field.

Beyond making sure that we put the user, and their quality of life, at the center of the process, it is also vital that we increase the degree of collaboration between researchers in the USA and Europe. This, in part, will serve to reduce the duplication of effort as we can see evidence of common activities in both regions. On the positive side, greater collaboration and communication can deliver a more efficient research and innovation process by means of achieving economies of scale, and enhanced combinations of expertise addressing the problem. It can also help to address the cultural perspective that can influence success in the field. Though the drive for increased collaboration needs to start with the researchers themselves, it can only be achieved on a wider scale by means of collaboration between the respective funding organizations in Europe and the USA. We need to establish common priority areas based on current unmet clinical and societal needs, develop roadmaps designed to address these needs, and finally create new funding models that will bring the optimal combination of expertise and infrastructure in Europe and the USA to bear on the area. Achieving this is not easy, but the potential benefits should justify the effort involved.

Finally, it is important to place the papers published in this special issue in context. As they are based on an NSF-sponsored visit, they do not follow the usual paper format that you might expect. The papers are a mix of commentaries and short reports/reviews of ongoing research activities in the areas covered during the visit. We have commentaries on European trends in mobility research and the need for improvements in this field. Alongside this we have review reports on trends in assistive technologies for mobility, technologies for enhancing movement training, personalized neuromusculoskeletal modeling, and wearable sensors as applied to rehabilitation. Finally, the delegation have reported on organizational structures promoting research, training and technology transfer in mobility technology research in Europe.

## Conclusion

In a recent presentation by the President of the United States, he stated that "Americans with disabilities are Americans first and foremost, and like all Americans are entitled to not only full participation in our society, but also full opportunity in our society." [5] The mobility technologies that are presented in this special edition of the Journal of NeuroEngineering and Rehabilitation will facilitate this greater societal participation by persons with disabilities and, ultimately, greater access to the opportunities that are available to all Americans, as envisioned by the President. As noted previously, the following papers represent the state-of-the-art in assistive devices for persons with mobility disabilities. We trust that you will derive as much enjoyment from reading these papers as we have had in bringing them to you.
